# A Virtual Force Algorithm-Lévy-Embedded Grey Wolf Optimization Algorithm for Wireless Sensor Network Coverage Optimization

**DOI:** 10.3390/s19122735

**Published:** 2019-06-18

**Authors:** Shipeng Wang, Xiaoping Yang, Xingqiao Wang, Zhihong Qian

**Affiliations:** College of Communication Engineering, Jilin University, Changchun 130012, China; spwang17@mails.jlu.edu.cn (S.W.); yxp@jlu.edu.cn (X.Y.); xqwang18@mails.jlu.edu.cn (X.W.)

**Keywords:** wireless sensor network, Lévy-embedded Grey Wolf Optimization algorithm, Virtual Force algorithm, coverage optimization

## Abstract

The random placement of a large-scale sensor network in an outdoor environment often causes low coverage. In order to effectively improve the coverage of a wireless sensor network in the monitoring area, a coverage optimization algorithm for wireless sensor networks with a Virtual Force-Lévy-embedded Grey Wolf Optimization (VFLGWO) algorithm is proposed. The simulation results show that the VFLGWO algorithm has a better optimization effect on the coverage rate, uniformity, and average moving distance of sensor nodes than a wireless sensor network coverage optimization algorithm using Lévy-embedded Grey Wolf Optimizer, Cuckoo Search algorithm, and Chaotic Particle Swarm Optimization. The VFLGWO algorithm has good adaptability with respect to changes of the number of sensor nodes and the size of the monitoring area.

## 1. Introduction

Wireless sensor networks (WSNs) were originally designed for military applications, however currently WSNs are also widely used in civilian applications, including vehicle tracking, forest monitoring, seismic observation, building monitoring, and water resource monitoring [1,2]. The coverage rate is an important measure of WSN performance. How to use the minimum number of sensor nodes to monitor the target area has been a question of particular interest regarding WSN technology. Wireless sensor nodes are typically placed randomly in the monitored area, which results in a non-uniform distribution of sensor nodes and a low coverage rate of the monitored area. Therefore, it is of great significance to improve the coverage rate of WSNs in the monitored area by adjusting the position of sensor nodes.

In 1999, the concept of swarm intelligence was formally proposed. Algorithms and solutions for a distributed problem inspired by the collective behavior of social insects and animal groups are part of swarm intelligence research. This type of algorithm is called a swarm intelligence optimization algorithm. Among intelligent algorithms, the Taguchi-teaching learning-based optimization algorithm and Jaya algorithm are also widely used [3,4]. In 2009, Yang and Deb of Cambridge University proposed a new biological heuristic algorithm, namely the Cuckoo Search algorithm [5]. This algorithm is widely used because of its simple structure, few control parameters, and strong search capability. Thanh-Phong Dao [6] proposed a hybrid Taguchi-Cuckoo Search (HTCS) to be applied to compatible focus positioning platforms (CFPP). The proposed HTCS approach can effectively optimize multiple objectives for the CFPP, and would be a useful technique for related optimization problems.

In recent years, scholars have carried out a lot of research on the GWO (Grey Wolf Optimizer). Heidari combined the GWO algorithm with Levi’s flight to propose the Lévy-embedded GWO (LGWO) [7]. This algorithm has a stronger search ability than the GWO. At the same time, Heidari has set up multiple sets of experiments to prove that the LGWO algorithm is better than PSO (Particle Swarm Optimization), CS (Cuckoo Search), DE (Differential Evolution), and other algorithms in most cases [7]. Therefore, the LGWO algorithm is applied to wireless sensor node deployment in this paper. The application of the LGWO algorithm to wireless sensor node deployment improves the coverage rate of the wireless sensor network. However, the sensor nodes are still non-uniform. In addition, many studies lack a design for how to complete wireless sensor node deployment with the shortest moving distance.

This paper proposes a wireless sensor network coverage optimization algorithm, the Virtual Force-Lévy-embedded Grey Wolf Optimization (VFLGWO) algorithm, which is intended to obtain a higher coverage rate of the monitoring area, a more uniform sensor nodes distribution, and a shorter average moving distance of the wireless sensor nodes. Typical performance evaluation indicators, such as the coverage rate, uniformity, average moving distance of the sensor nodes, and running time are simulated. Compared with wireless sensor network coverage optimization algorithms using Chaos Particle Swarm Optimization (CPSO), Cuckoo Search (CS) and Lévy-embedded Grey Wolf Optimization (LGWO), the simulation experiment proves the performance superiority of the VFLGWO.

The contributions of this paper are:

1. This paper applies the LGWO algorithm to wireless sensor node deployment.
2. For wireless sensor node deployment, this paper proposes the VFLGWO algorithm, which achieves a higher coverage rate.
3. This paper designs sensor node matching algorithms to make the average moving distance of sensor nodes shorter.

The remainder of this paper is structured as follows. Section 2 describes the related work. Section 3 describes the proposed VFLGWO based sensor deployment scheme. The simulation results and comparison of the CPSO algorithm, CS algorithm, and LGWO algorithm are presented in Section 4, and conclusions are drawn in Section 5.

## 2. Related Work

### 2.1. Research Situation

In Guanglin Zhang’s work [8], Guanglin Zhang and Shan You studied the coverage of WSNs. They employed a Voronoi diagram to address the coverage problem. The authors transformed the network area coverage problem into cell coverage problems with the Voronoi diagram, which only needs to optimize local coverage for each cell in a decentralized way.

The research on using swarm intelligence optimization algorithms to solve WSN coverage optimization problems is gradually increasing. Ning-ning Qin and Leopoldo Eduardo Cárdenas-Barrón [9,10] applied differential evolution and its improved algorithm to complete wireless sensor node deployment. Differential evolution has the characteristics of a simple structure, easy implementation, fast convergence, and strong robustness. Jia, Jie [11] adopted a genetic algorithm to solve the WSN coverage optimization problem. Genetic algorithms have a better ability to conduct a global search, but their implementation is relatively complex and their convergence speed is slow. The PSO algorithm [12,13,14] has been applied to the deployment of wireless sensor networks by scholars. The PSO is easy to implement and can effectively balance the directionality, diversity, and balance in the search. In other research [15], the PSO algorithm was employed to solve the optimization problem for a compliant rotary joint for an upper limb assistive device, and obtained a CRJ with a smaller error. However, the PSO algorithm has some shortcomings, including relying too much on search parameters, easily falling into local extremum, and premature convergence. In Celal Ozturk’s work [16], an artificial bee colony algorithm was used to maximize area coverage in WSNs. The artificial bee colony algorithm has the advantage of easy realization, and the shortcoming of easily falling into the local solution. Some scholars have adopted the Chaotic Artificial Fish Swarm algorithm to solve this problem [17]. The advantage of the Artificial Fish Swarm algorithm is that it has better robustness. The algorithm adopts an adaptive step size, which effectively increases the coverage of the algorithm.

### 2.2. An Overview of the GWO Algorithm

Inspired by wolf hunting behavior, Mirjalili et al. proposed the Grey Wolf Optimization (GWO) [18], which is easy to implement because of its few control parameters. The GWO algorithm is inspired by the social hierarchy and hunting strategies of grey wolves in the wild. Each grey wolf represents a potential solution to the population. The position of *α* wolf, which is the leading wolf, is the best position. The position of the *β* wolf in the second class of the wolf rank, and the *δ* wolf responsible for reconnaissance, vigilance, warding and guarding, are the second-best solution and the third best solution, respectively. The other candidate solutions are the lower-level *ω* wolf positions. In the conventional GWO, the motion of wolves is described as follows.

#### 2.2.1. Population Initialization

Since the performance of the GWO is less affected by the initial value of the population, it is initialized in the algorithm by randomly generating:

(1) $$X_{i,j}∼U\left(lb_{j},ub_{j}\right)$$

where *X* is the grey wolf population, *i* ∈ [1, 2, 3…, *N*] and *j* ∈ [1, 2, 3…, *sizepop*], *N* is the number of grey wolf populations, *sizepop* is the population dimension; *lb* and *ub* are the lower bound and upper bound of the search interval respectively; and *U* is a random uniform distribution function.

#### 2.2.2. Encircling Prey

As mentioned above, grey wolves encircle prey during the hunt. In order to mathematically model encircling behavior, the following equations are proposed:

(2) $$\vec{D}=\left|\vec{C}\cdot {\vec{X}}_{p}\left(t\right)-\vec{X}\left(t\right)\right|$$

(3) $$\vec{X}\left(t+1\right)={\vec{X}}_{p}\left(t\right)-\vec{A}\cdot \vec{D}$$

where $\vec{D}$ is the distance between the prey and grey wolf; *t* indicates the current iteration, $\vec{A}$ and $\vec{C}$ are coefficient vectors, ${\vec{X}}_{p}$ is the position vector of the prey; and $\vec{X}$ indicates the position vector of a grey wolf.

The vectors $\vec{A}$ and $\vec{C}$ are calculated as follows:

(4) $$\vec{A}=2\cdot \vec{a}\cdot {\vec{r}}_{1}-\vec{a}$$

(5) $$\vec{C}=2\cdot {\vec{r}}_{2}$$

where components of $\vec{a}$ are linearly decreased from 2 to 0 over the course of iterations, and ${\vec{r}}_{1}$, ${\vec{r}}_{2}$ are random vectors in [0,1]. Candidate solutions tend to diverge from the prey when $\left|\vec{A}\right|\geq 1$ and converge towards the prey when $\left|\vec{A}\right|<1$.

#### 2.2.3. Population Location Update

By calculating the objective function value of the GWO algorithm, the best solution, the second best solution, and the third best solution are set to *α* wolf, *β* wolf, and *δ* wolf. The position of other grey wolves is determined by the position of *α* wolf, *β* wolf and *δ* wolf, as shown in Equations (6)–(8). After generating a new group, boundary control is performed on the elements in the population to complete an iteration. The above process is repeated until the algorithm termination condition is satisfied, and finally the optimal solution is outputted.

(6) $$\{\begin{array}{c} {\vec{D}}_{\alpha }=\left|{\vec{C}}_{1}\cdot {\vec{X}}_{\alpha }-\vec{X}\right|\\ {\vec{D}}_{\beta }=\left|{\vec{C}}_{2}\cdot {\vec{X}}_{\beta }-\vec{X}\right|\\ {\vec{D}}_{\delta }=\left|{\vec{C}}_{3}\cdot {\vec{X}}_{\delta }-\vec{X}\right| \end{array}$$

(7) $$\{\begin{array}{c} {\vec{X}}_{1}={\vec{X}}_{\alpha }-{\vec{A}}_{1}\cdot \left({\vec{D}}_{\alpha }\right)\\ {\vec{X}}_{2}={\vec{X}}_{\beta }-{\vec{A}}_{2}\cdot \left({\vec{D}}_{\beta }\right)\\ {\vec{X}}_{3}={\vec{X}}_{\delta }-{\vec{A}}_{3}\cdot \left({\vec{D}}_{\delta }\right) \end{array}$$

(8) $$\vec{X}\left(t+1\right)=\frac{{\vec{X}}_{1}+{\vec{X}}_{2}+{\vec{X}}_{3}}{3}$$

### 2.3. Lévy-Embedded Grey Wolf Optimization Algorithm

The GWO algorithm has the advantages of not relying on parameter settings and convenient implementation, however the GWO algorithm is still prone to prematurely falling into local extremum when solving complex optimization problems; that is, the phenomenon of premature convergence occurs. In order to alleviate the above problems, Heidari proposed a Lévy-embedded Grey Wolf Optimization (LGWO) algorithm based on the GWO algorithm combined with Lévy Flight [7]. The Lévy Flight can assist the GWO in searching based on deeper searching patterns. Using this concept, it can be ensured that the GWO can handle global searching more efficiently. The LGWO algorithm mainly improves the GWO algorithm in three aspects.

1. The role of *δ* wolves in the social hierarchy will be played by other wolves.
2. The Lévy Flight concept is embedded into the modified GWO.
3. The greedy selection (GS) strategy is employed for the LGWO.

The specific process of the LGWO algorithm is summarized as follows, and the pseudo code of the LGWO algorithm is described in detail, as shown in Table 1.

1. Population initialization. The process is the same as Equation (1) of Section 2.2.1 for the GWO algorithm.
2. Population search. The process is the same as Equations (2) and (3) of the GWO algorithm in Section 2.2.2.
3. By calculating the objective function value of the LGWO algorithm, the best solution *α* wolf and second-best solution *β* wolf are obtained. The positions of other grey wolves are determined by the position of *α* wolf and *β* wolf, as shown in Equation (9):
  (9) $$\vec{X}\left(t+1\right)=\{\begin{array}{c} 0.5\times \left(\vec{X_{\alpha }}-\vec{A_{1}}\vec{D_{\alpha }}+\vec{X_{\beta }}-\vec{A_{2}}\vec{D_{\beta }}\right)+\alpha ⊕Levi\left(\beta \right)\;\left|A\right|\geq 0.5\\ 0.5\times \left(\vec{X_{\alpha }}-\vec{A_{1}}\vec{D_{\alpha }}+\vec{X_{\beta }}-\vec{A_{2}}\vec{D_{\beta }}\right)\;\;\;\;\;\left|A\right|<0.5 \end{array}$$
  where
  (10) $$\alpha ⊕Levi\left(\beta \right)\;∼0.01\frac{u}{v^{-\beta }}\left(\vec{X}\left(t\right)-{\vec{X}}_{\alpha }\left(t\right)\right)$$
  where *u* and *v* follow a normal distribution:
  $$u∼N\left(0,\sigma_{u}^{2}\right),v∼N\left(0,\sigma_{v}^{2}\right)$$
  $$\sigma_{u}={\left[\frac{\Gamma \left(1+\beta \right)\sin(\frac{\pi \beta }{2})}{\Gamma \left(\frac{1+\beta }{2}\right)\beta \times 2^{\frac{\beta -1}{2}}}\right]}^{\frac{1}{\beta }},\sigma_{v}=1$$

In the LGWO Algorithm 1 proposed by Heidari, the parameter *β* is a random number of [0,2].

**Algorithm 1** Pseudo code for the LGWO algorithm
Determine the initial swarm size *N* and number of iterations *T*Randomly generate the initial population of wolvesInitialize *a*, *p*, $\vec{A},\;\vec{C}$Compute the fitness of each wolfSet to ${\vec{X}}_{\alpha }$ be the best wolfSet to ${\vec{X}}_{\beta }$ be the second best wolf**While** (*t* < *T*) or (stopping condition) **do**  **For** each wolf  Update the position of current wolves by Equation (13)  Perform GS by Equation (15)  **end for**Update *a*, *p*, $\vec{A},\;\vec{C}$Calculate the fitness of wolvesUpdate ${\vec{X}}_{\alpha }$ and ${\vec{X}}_{\beta }$*t* = *t* + 1
**end while**
Return ${\vec{X}}_{\alpha }$


After generating a new group, Heidari [7] selected whether to retain the updated grey wolf through the greedy selection strategy depicted by Equation (11), and completes an iteration. The above process is repeated until the algorithm termination condition is satisfied, and finally the optimal solution is outputted.

(11) $$\vec{X}\left(t+1\right)=\{\begin{array}{c} \vec{X}\left(t\right)f\left({\vec{X}}_{new}\left(t\right)\right)>f\left(\vec{X}\left(t\right)\right)\mathrm{and}\;r_{new}<p\\ {\vec{X}}_{new}\left(t\right)\;\;\;\;\;\;\;\;\;\mathrm{otherwise} \end{array}$$

where *r_new_* and *p* are random values inside (0, 1).

Additionally, referring to the greedy selection (GS) strategy from the DE algorithm, the concept of “survival of the fittest” is utilized here with the probability *p*. According to this strategy, new superior positions in each generation can continue to be more enriched for the next generations and the worse ones are disregarded, so the LGWO algorithm has a stronger search capability.

### 2.4. Virtual Force Algorithm

The Virtual Force Algorithm (VFA) was originally an algorithm that allowed mobile robots to evade obstacles in an unknown environment [19,20,21]. The VFA has been applied to WSN coverage optimization, and can quickly disperse sensor nodes. The VFA treats the sensor nodes as charged particles. The forces between the sensor nodes are presented by attractive and repulsive patterns. When two nodes are close enough, smaller than the threshold *D_th_*, the force is in a repulsive pattern, which intends to separate them. When the distance between two nodes is larger than the threshold *D_th_*, the force becomes an attractive pattern, which draws them closer. Assuming that the set of sensor nodes in the region is *S* = {*s*_1_, *s*_2_, *s*_3_, …, *s_l_*}, then the distance *d_ij_* between the *i*, *j* sensor nodes is *s_i_* (*x_i_*, *y_i_*) and *s_j_* (*x_j_*, *y_j_*):

(12) $$d_{ij}=d\left(s_{i},s_{j}\right)=\sqrt{{\left(x_{i}-x_{j}\right)}^{2}+{\left(y_{i}-y_{j}\right)}^{2}}$$

Then, the force of the sensor node *s_j_* on the sensor node *s_i_* is:

(13) $${\vec{F}}_{ij}=\{\begin{array}{c} \omega_{\alpha }\left(d_{ij}-D_{th}\right),\alpha_{ij}\;\;d_{ij}>D_{th}\\ 0\;\;\;\;\;\;\;d_{ij}=D_{th}\;\\ \omega_{\gamma }/d_{ij},\alpha_{ij}+\pi \;\;\;d_{ij}<D_{th} \end{array}$$

where $\omega_{\alpha }$ and $\omega_{\gamma }$ are the virtual force coefficients, $\omega_{\gamma }\gg \omega_{\alpha }$; *D_th_* is the threshold for generating gravitation and repulsive force, and the value of *D_th_* is $\sqrt{3}R_{s}$ [20]; and $\alpha_{ij}$ is the angle of the line between sensor node *i* and sensor node *j* with respect to the y-axis. If *d_ij_* < *D_th_*, it is considered that sensor node *i* is too close to sensor node *j*, and the force is in a repulsive pattern; if *d_ij_* > *D_th_*, the force becomes an attractive pattern. The resultant force of the sensor node *i* is ${\vec{F}}_{i}$, the resultant force in the horizontal direction is ${\vec{F}}_{ix}$, the resultant force in the vertical direction is ${\vec{F}}_{iy}$, and the position of the node is updated by Equations (14) and (15).

(14) $$x\left(k+1\right)=x\left(k\right)+\frac{{\vec{F}}_{ix}}{{\vec{F}}_{i}}\times MaxStep\times e^{-1/{\vec{F}}_{i}}$$

(15) $$y\left(k+1\right)=y\left(k\right)+\frac{{\vec{F}}_{iy}}{{\vec{F}}_{i}}\times MaxStep\times e^{-1/{\vec{F}}_{i}}$$

where *x*(*k*) and *y*(*k*) are the horizontal and vertical coordinates of sensor node *s_i_* at time *k*, respectively; and *MaxStep* is the step size for the wireless sensor node to move.

### 2.5. Improved Virtual Force Algorithm

Generally, the communication radius *R* of the sensor node is defined as twice the perceived radius *R_s_*. Actually, when *d_ij_* is larger than *R*, they cannot exchange information with each other, they cannot calculate or obtain the distance, and the VFA cannot be used in this case. The purpose of applying the improved virtual force algorithm in this paper is to apply it to wireless sensor network node deployment, and specifically to apply it to wireless sensor network nodes, which not only have sensor sensing capabilities, but also have communication capabilities. Therefore, the distance between sensor nodes must be less than the communication radius, and the sensor node distribution should not be too dense.

In the improved virtual force algorithm applied in this paper, when the *d_ij_* is greater than *R*, there is no virtual force between the sensor nodes. Equation (13) of the sensor node *s_j_* to the sensor node *s_i_* is modified:

(16) $${\vec{F}}_{ij}=\{\begin{array}{c} \omega_{\alpha }\left(d_{ij}-D_{th}\right),\alpha_{ij}\;\;R\geq d_{ij}>D_{th}\\ 0\;\;\;\;\;\;\;d_{ij}=D_{th}\;\mathrm{or}\;d_{ij}>R\;\\ \omega_{\gamma }/d_{ij},\alpha_{ij}+\pi \;\;\;d_{ij}<D_{th} \end{array}$$

The physical meaning of the variables and parameters in Equation (16) is the same as Equation (13), and the updated equation of the sensor node position is the same as Equations (14) and (15).

## 3. Wireless Sensor Node Deployment with the VFLGWO Algorithm

### 3.1. Problem Description

#### 3.1.1. Network Model

1. All sensor nodes are mobile.
2. All sensor nodes are omnidirectional sensors, and the perceptual model is a probabilistic detection model.
3. All sensor node location information is known by GPS.
4. All sensor nodes are able to move to the scheduled position, where the scheduled position is within their mobility range.

#### 3.1.2. Coverage Rate

**Figure 1:**
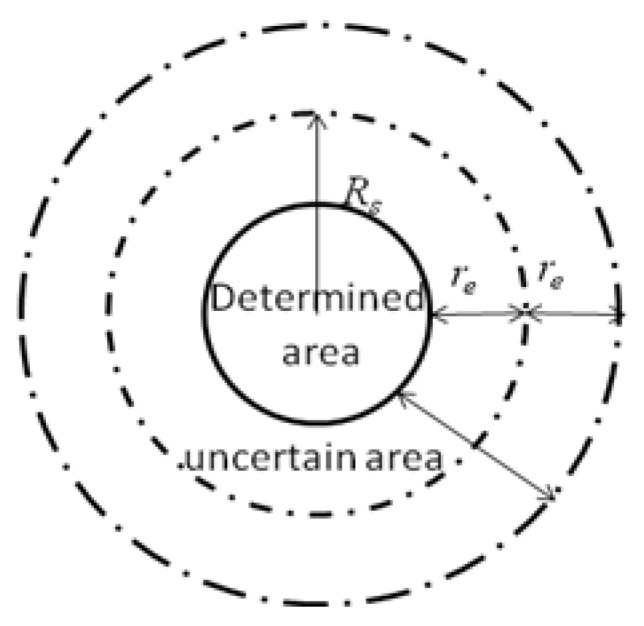
Node sensing model.

The sensor node in this paper adopts the probabilistic detection model. Compared with the disk model, this model can objectively reflect the real network deployment environment, as shown in Figure 1. In Figure 1, the inside of the solid circle is the determined area, and the area between the solid circle and the outermost dotted circle is an uncertain area. The outside of the outermost dotted circle is an unmeasured area. Assuming that the monitoring area is a two-dimensional planar area, each sensor node can be deployed anywhere within the area. If the coordinates of the sensor node *s_i_* in the sensor network are (*x_i_*, *y_i_*) and the coordinates of the monitoring target point *p* (pixel point) in the region are (*x_p_*, *y_p_*), then the monitoring probability *C_p_* (*s_i_*, *p*) of the sensor node *s_i_* to the target point *p* is set as follows:

(17) $$C_{p}\left(s_{i},p\right)=\{\begin{array}{c} 1\;\;\;\;\;\;d\left(s_{i},p\right)\leq R_{s}-r_{e}\\ \exp\left(\frac{-\lambda_{1}\alpha_{1}^{\beta_{1}}}{\alpha_{2}^{\beta_{2}}}+\lambda_{2}\right)R_{s}-r_{e}<d\left(s_{i},p\right)<R_{s}+r_{e}\\ 0\;\;\;\;\;\;\;otherwise \end{array}$$

where *d*(*s_i_*,*p*) is the Euclidean distance between the sensor node *s_i_* and the target point *p*; *R_s_* is the distance radius that the sensor node can perceive; *r_e_*(0 < *r_e_* < *R_s_*) is the measurement reliability parameter of the sensor node; *α*_1_ = *r_e_* − *R_s_* + *d*(*s_i_*,*p*), *α*_2_ = *r_e_* + *R_s_* − *d*(*s_i_*,*p*), and *α*_1_ > 0, *α*_2_ > 0; *λ*_1_, *λ*_2_, *β*_1_, *β*_2_ are the measurement parameters related to the characteristics of the sensor node. The parameters of the work by Author [22] are used to set *λ*_1_ = 1, *λ*_2_ = 0, *β*_1_ = 1, *β*_2_ = 1.5.

Let the probability threshold of target point *p* be detected as *C_th_*, and the joint monitoring probability *C_p_*(*s_all_*, *p*) of the sensor node to target point *p* in the whole monitoring area is:

(18) $$C_{p}\left(s_{all},p\right)=\{\begin{array}{c} 1\;,\;\left(1-\overset{l}{\underset{i=1}{\prod }}\left[1-C_{p}\left(s_{i},p\right)\right]\right)\geq C_{th}\\ 0\;,\;\left(1-\overset{l}{\underset{i=1}{\prod }}\left[1-C_{p}\left(s_{i},p\right)\right]\right){\;<\;C}_{th} \end{array}$$

where *s_all_* represents the set of sensor nodes that measure target point *p*, and *l* is the number of sensor nodes in the monitoring area.

Assuming that the monitoring area is a rectangle of *m* × *n* (m^2^), the area to be measured is divided into *m* × *n* identical grids of equal size and area of 1 m^2^. Then, the grids are simplified to pixel points with a discrete precision of 1. In this paper, the WSN node coverage rate is defined as the ratio of the number of grids covered by Equation (19) to the total number of grids in the monitoring area, namely:

(19) $$fitness=\frac{\sum_{x_{p}=1}^{m}\sum_{y_{p}=1}^{n}C_{p}\left(s_{all},p\right)}{m\times n}$$

Then, the coverage optimization problem is briefly described as follows:

Step 1. Calculate the probability of monitoring one-pixel point by one sensor node using Equation (17).
Step 2. Calculate the joint monitoring probability of all sensor nodes to one-pixel point using Equation (18).
Step 3. The coverage rate of the monitored area is calculated using Equation (19). Equation (19) is used as the objective function of the coverage optimization algorithm.

#### 3.1.3. Uniformity of Sensor Node Distribution

Uniformity is an important indicator for measuring the uniform distribution of sensor nodes in wireless sensor networks. It is used to describe the uniformity of the sensor node distribution. It is usually expressed by the mean of the sum of the standard deviation of all sensor nodes. The wireless sensor network node uniformity indicator *E* is calculated as shown in Equation (20):

(20) $$E=\frac{1}{l}\overset{l}{\underset{i=1}{\sum }}E_{i}\;;\;E_{i}={\left[\frac{1}{k}\overset{k}{\underset{j=1}{\sum }}{\left(D_{i,j}-M_{i}\right)}^{2}\right]}^{\frac{1}{2}}$$

where *l* is the total number of sensor nodes, *k* is the number of neighboring nodes of sensor node *i*, *D_i,j_* is the Euclidean distance between adjacent nodes *i*, *j*, and *M_i_* is the average of the Euclidean distance between the sensor node *i* and all its neighboring sensor nodes. It can be found from Equation (20) that the closer the values of *D_i,j_* and *M_i_* are, the more uniform the distribution is. Therefore, the smaller the *E* value, the better the uniformity of the sensor node distribution.

#### 3.1.4. Average Moving Distance of Sensor Nodes

The average moving distance of sensor nodes refers to the average distance that each sensor node in the wireless sensor network moves from the initial position to the best position with the largest final coverage rate. It is assumed that the initial position information of each sensor node is known. Through the MATLAB simulation, the VFLGWO algorithm is used to solve the optimal position of each sensor node with the largest coverage rate. Each sensor node is directly moved from the initial position to the optimal position in a straight line. Obviously, for wireless sensor nodes, the shorter the average moving distance is, the smaller the energy consumption is.

### 3.2. The VFLGWO Algorithm

#### 3.2.1. The Modified LGWO Algorithm

The LGWO algorithm applies GS strategy to allow the algorithm to retain a better solution for each iteration. For the LGWO algorithm, $f\left(\vec{X}\right)$ represents a convergence curve and the smaller the value, the better. However, for wireless sensor node deployment, $f\left(\vec{X}\right)$ represents $fitness\left(\vec{X}\right)$ (the coverage rate). The larger the $fitness\left(\vec{X}\right)$ value that is obtained, the larger the corresponding coverage rate. Therefore, the modified LGWO proposed in this paper is the same as the LGWO, except that Equation (11) is modified to Equation (21). When the new value $fitness\left(\vec{X}\right)$ obtained by a new iteration is not greater than the previous generation value and the satisfaction probability condition is reached, the optimal solution is not updated; otherwise, the obtained superior solution is substituted for the previous generation solution.

(21) $$\vec{X}\left(t+1\right)=\{\begin{array}{c} \vec{X}\left(t\right)f\left({\vec{X}}_{new}\left(t\right)\right)<f\left(\vec{X}\left(t\right)\right)\;\mathrm{and}\;r_{new}<p\\ {\vec{X}}_{new}\left(t\right)\;\;\;\;\;\;\;\;\mathrm{otherwise} \end{array}$$

#### 3.2.2. Combination of the Improved Virtual Force Algorithm and the Modified LGWO Algorithm

The performance of the LGWO is significantly better than that of the GWO. However, when the modified LGWO algorithm is applied to WSN node deployment, the problem of non-uniform distribution of sensor nodes still exists, while the Virtual Force algorithm can make the sensor nodes more uniform. Therefore, combined with the modified LGWO algorithm, the improved Virtual Force algorithm is introduced. In this paper, the Virtual Force-Lévy-embedded Grey Wolf Optimization (VFLGWO) algorithm is proposed.

In the modified LGWO algorithm, the position of a wolf represents a possible solution to the optimization problem. Therefore, the deployment of the sensor nodes in the detection area refers to a wolf (a solution) in the algorithm.

The procedure of deploying wireless sensor nodes by the VFLGWO algorithm can be summarized as follows:

*N* sensor nodes are randomly generated to be the initial position of the real sensor nodes. Initialize each wolf (each set of solutions) by the modified LGWO algorithm as the virtual sensor node position.

Enter the iteration, update the position of each wolf using Equations (9) and (21). Then, apply the improved Virtual Force algorithm to update the sensor nodes’ positions for each wolf, calculate the virtual force using Equation (20), and adjust the position of the nodes for each wolf using Equations (14) and (15). Apply the survival of the fittest selection rule using Equation (22), and retain the better solution obtained by the improved virtual force algorithm:

(22) $$\vec{X}\left(t+1\right)=\{\begin{array}{c} \vec{X}\left(t\right)\;\;f\left({\vec{X}}_{new}\left(t\right)\right)<f\left(\vec{X}\left(t\right)\right)\\ {\vec{X}}_{new}\left(t\right)\;\;\;\;\;\;\mathrm{otherwise} \end{array}$$

Through the superior solution of the *t*_th_ generation $\vec{X}\prime \left(t\right)$, whether to update *α* wolf or *β* wolf is determined; the VFLGWO algorithm completes an iteration. If the number of iterations does not reach the specified number of iterations *T*, perform the above iterative process again; otherwise, the process ends. The specific implementation of the VFLGWO algorithm will be described in Section 3.3.

### 3.3. Procedure for the VFLGWO Algorithm

**Figure 2:**
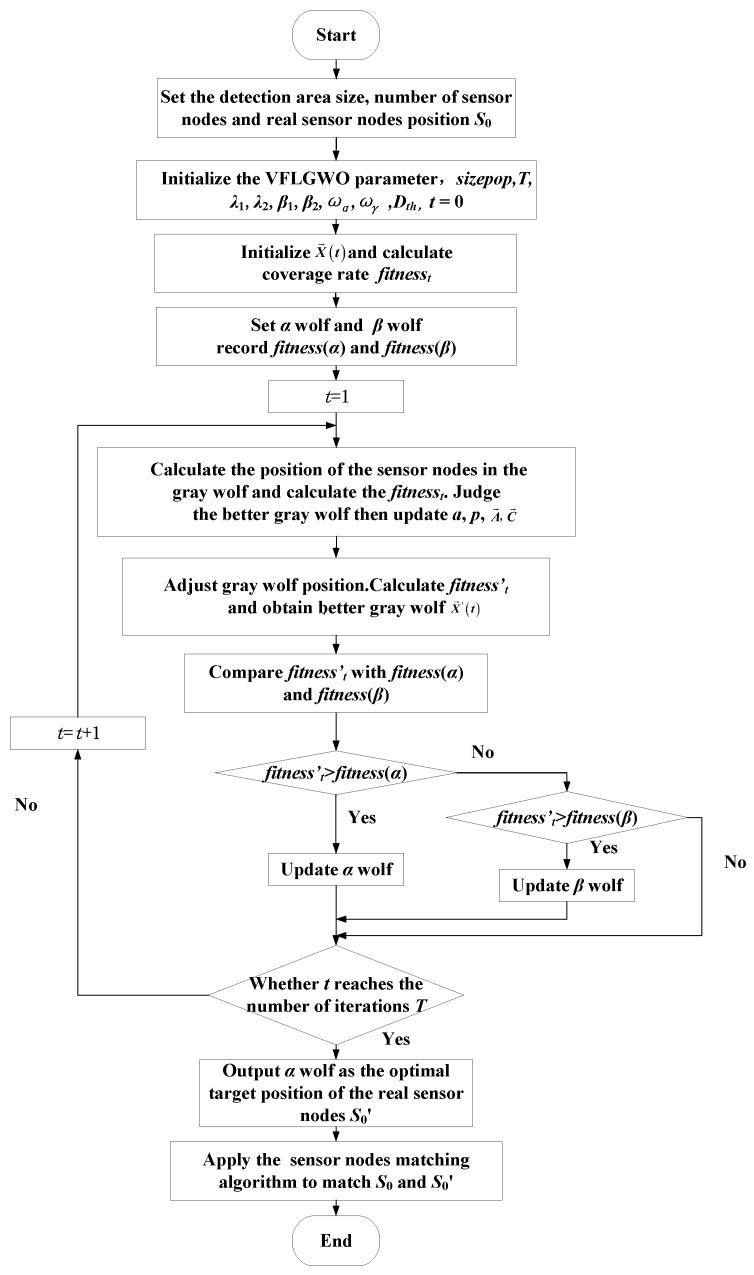
Process of the VFLGWO algorithm.

Application of the VFLGWO algorithm in WSN node deployment is shown in Figure 2. The steps of the VFLGWO algorithm are described as follows:

1. Determine the size of the monitoring area and the number of sensor nodes *N* to be deployed, and randomly deploy the sensor nodes in the monitoring area at the real sensor node positions, the set of which is *S*_0_ = {*s*_1_, *s*_2_, *s*_3_, …, *s_N_*}.
2. Initialize the parameters of the VFLGWO algorithm: population size *sizepop*, iteration number *T*, related parameters of the improved virtual force algorithm $\omega_{\alpha }$, $\omega_{\gamma }$ and *D_th_*, and related parameters of the probability perception model *λ*_1_, *λ*_2_, *β*_1_, *β*_2_; *t* = 1.
3. Generate the initialized grey wolf population $\vec{X}\left(t\right)$ randomly and calculate the coverage rate *fitness_t_* of each wolf (each set of solutions) using Equations (17)–(19).
4. Set the solution with the largest coverage rate to *α* wolf, set the solution with the second largest coverage rate to *β* wolf, and record the coverage rate *fitness* (*α*) and *fitness* (*β*) of *α* wolf and *β* wolf.
5. Enter the iteration, set *t* = 1, calculate the position of each grey wolf after the *t*th iteration using Equation (11), calculate the coverage *fitness_t_* of each wolf, judge the better grey wolf using Equation (21), and then update *a*, *p*, $\vec{A},\;\vec{C}$.
6. Apply the improved virtual force algorithm to calculate the virtual force of each sensor node of each wolf using Equation (16), adjust the position of each sensor node of each wolf using Equations (14) and (15), calculate the coverage *fitness_t_’* of each wolf, and obtain better grey wolves $\vec{X}\prime \left(t\right)$ using Equation (22).
7. Compare the *fitnesst’* value of the *t*th iteration with the *fitness*(*α*) and *fitness*(*β*) obtained by the previous iteration to determine whether to update the α wolf or the *β* wolf.
8. Determine whether the number of iterations reaches the specified number of iterations *T*, “No” then *t* = *t* + 1, return 5); “Yes” then the iteration ends.
9. Output the optimal solution *α* wolf as the optimal target position of the real sensor nodes, recorded as $S_{0}{}^{\prime }=\left\{S_{1}{}^{\prime },S_{2}{}^{\prime },S_{3}{}^{\prime },\ldots ,S_{N}{}^{\prime }\right\}$.
10. Use the sensor nodes matching algorithm, and match the position of the initial sensor node positions *S*_0_ = {*s*_1_, *s*_2_, *s*_3_, …, *s_N_*} and the target position of the optimal solution $S_{0}{}^{\prime }=\left\{S_{1}{}^{\prime },S_{2}{}^{\prime },S_{3}{}^{\prime },\ldots ,S_{N}{}^{\prime }\right\}$ to complete the deployment of the WSN.

There are currently two options for WSN node deployment: (1) give the target sensor node position without considering how the sensor nodes move; (2) match the same sequence numbers of the sensor nodes before and after optimization and design the corresponding relationship of node movements.

The pseudo code of the sensor nodes matching algorithm proposed in this paper is shown in Algorithm 2. The initial position set of *N* sensor nodes in the region is *S*_0_ = {*s*_1_, *s*_2_, *s*_3_, …, *s_N_*}, and the target position for obtaining the optimal solution is $S_{0}{}^{\prime }=\left\{S_{1}{}^{\prime },S_{2}{}^{\prime },S_{3}{}^{\prime },\ldots ,S_{N}{}^{\prime }\right\}$. The matching of *S*_0_ and $S_{0}{}^{\prime }$ is achieved by the sensor nodes matching algorithm.

**Algorithm 2** Pseudo code for sensor nodes matching algorithm
Input initial position set *S*_0_, optimal solution position set *S*_0_′, number of nodes *N***For***i* from 1 to *N* **do**  **For** *j* from 1 to *N* **do**   Calculate the Euclidean distance between the *S*_0_ node position *s_i_* and the *S*_0_′ node position *s_j_* and record  **end for**
**end for**
*k* = *N***While** (*k* > 0) and (*s_i_*′, *s_j_*′ are unmatched nodes in *S*_0_, *S*_0_′) **do**   Take the shortest distance between the shortest *s_i_* in *S*_0_ and *s_j_* in *S*_0_′ to complete position matching and record   *k* = *k* − 1
**end while**
Return the match result of *S*_0_, *S*_0_′


The VFLGWO algorithm proposed in this paper applies the LGWO algorithm with a stronger search ability to the deployment of wireless sensor nodes, and combines the improved Virtual Force algorithm to achieve a wireless sensor network with a higher coverage rate and more uniform distribution. In order to achieve the goal of making the average moving distance of the sensor nodes shorter, the randomly generated real node positions are taken as the initial positions of the sensor nodes, and the sensor node position of the optimal solution obtained by the VFLGWO algorithm is taken as the final position of the real node. The sensor nodes matching algorithm proposed in this paper is used to complete the WSN node deployment.

### 3.4. Algorithm Time Complexity Analysis

For the modified LGWO algorithm, initialize the number of sensor nodes N, and initialize the number of grey wolves m and the maximum number of iterations T. The modified LGWO algorithm calculates a wolf (update N sensor node position) with a time complexity of O(N). According to the LGWO algorithm, each iteration needs to update the position of the N sensor nodes of each wolf in the m wolf at one time. The time complexity of the algorithm to update the position at one iteration is O(m × N), so the time complexity required in the case of T iterations is O(T × m × N). Similarly, compared with the modified LGWO, the CPSO algorithm and CS algorithm only have different search methods, so the iterative process is similar. Therefore, the time complexity of the coverage strategy of the CPSO algorithm and CS algorithm is also O(T × m × N).

The VFLGWO algorithm proposed in this paper executes the improved Virtual Force algorithm again after the LGWO search phase. The time complexity of the improved Virtual Force algorithm is O (*N^2^*). Therefore, according to the VFLGWO algorithm process, it needs to be updated to *N* times per iteration. The time complexity is O (*m* × *N*^2^). In addition, the *T* set in this paper is much larger than *N*. Therefore, the time complexity of the sensor nodes matching algorithm proposed in this paper is negligible. The time complexity of the total coverage strategy of the VFLGWO algorithm is O (*T* ×*m* × *N*^2^).

Although the VFLGWO algorithm increases the amount of computation, the coverage rate and uniformity obtained by the VFLGWO algorithm are significantly higher than other algorithms. The position adjustment of the sensor nodes after random delivery is not very time-sensitive. The position-adjusted coverage rate is significant, therefore it makes sense to increase the amount of computation to achieve higher wireless sensor network deployment performance.

## 4. VFLGWO Algorithm Simulation

Firstly, the relevant parameters of the VFLGWO algorithm are discussed through simulation experiments. Different simulation experiments are designed to test the performance of the VFLGWO algorithm. The performance indexes of the simulation test in this paper include: coverage rate, uniformity, average moving distance of sensor nodes (m), and running time (s). When testing the average moving distance of sensor nodes, the VFLGWO algorithm applies the sensor nodes matching algorithm proposed in this paper. Other comparison algorithms still use the conventional method in which the *S*_0_ and $S_{0}{}^{\prime }$ are matched by the sequence number of sensor nodes.

In this paper, the experiments are performed using MATLAB 2014. The simulation environment is an area of 50 m × 50 m. The number of randomly distributed mobile sensor nodes is *N* = 50, and the sensing radius is *R_s_* = 5 m for all sensor nodes; the communication radius is *R* = 2*R_s_* = 10 m, the detection probability threshold is *C_th_* = 0.8, the measurement reliability parameter is *r_e_* = 2.5 m, the number of basic iterations is *T* = 3000 generations, and the population number is *sizepop* = 30 (the algorithm uses the cell array to store the sensor node position coordinates, and each wolf contains 50 sensor node position coordinates).

### 4.1. VFLGWO Algorithm Parameter Selection

#### 4.1.1. Determination of Gravity and Repulsion Parameters

According to the gravity and repulsion parameters of the VFLGWO algorithm, six groups of experiments are used to obtain relatively optimal $\omega_{\alpha }$ and $\omega_{\gamma }$. Each group of experiments is performed 20 times independently, and the average values of each indicator are obtained. The control variable method is applied. Firstly, the *MaxStep* is unified at 1.2 m, and the experimental value of $\omega_{\alpha }$ is unchanged at 1. The values of $\omega_{\gamma }$ are 600, 800, 1000, 1500 and 2000, respectively. Since this article mainly discusses how to deploy sensor nodes to maximize the coverage rate of the monitoring area, coverage rate is taken as the first indicator. The four optimization indicators calculated by the VFLGWO algorithm are listed in Table 1. As can be seen from Table 1, when the value of $\omega_{\gamma }$ is 1000, the coverage rate is the largest. Therefore, in the simulation experiment of the proposed algorithm, $\omega_{\alpha }$ is set to 1 and $\omega_{\gamma }$ is set to 1000.

#### 4.1.2. Determination of the *MaxStep* Parameter

Five groups of experiments are designed for the moving step *MaxStep* of the sensor nodes in the VFLGWO algorithm. Each group of experiments is performed 20 times, and the average value of each indicator is calculated. The *MaxStep* values are 0.6 m, 0.8 m, 1 m, 1.2 m and 1.4 m, respectively. The experimental results are shown in Table 2. When the *MaxStep* is 1.2 m, the coverage rate is the largest. Therefore, set the *MaxStep* to 1.2 m.

### 4.2. VFLGWO Algorithm Effectiveness Simulation Experiment

In order to verify the performance of the VFLGWO algorithm, the VFLGWO is compared with the wireless sensor network coverage optimization algorithm using Chaotic Particle Swarm (CPSO), Cuckoo Search (CS), and LGWO.

#### 4.2.1. Comparison Algorithm Parameter Settings

The parameters of the CPSO, CS, and LGWO are set as shown in Table 3 [7]. The other parameters, such as the size of the population *sizepop* and the number of basic iterations *T*, are the same as those of the VFLGWO algorithm.

#### 4.2.2. Deployments of the Sensor Positions after Optimization of Four Algorithms

**Figure 3:**
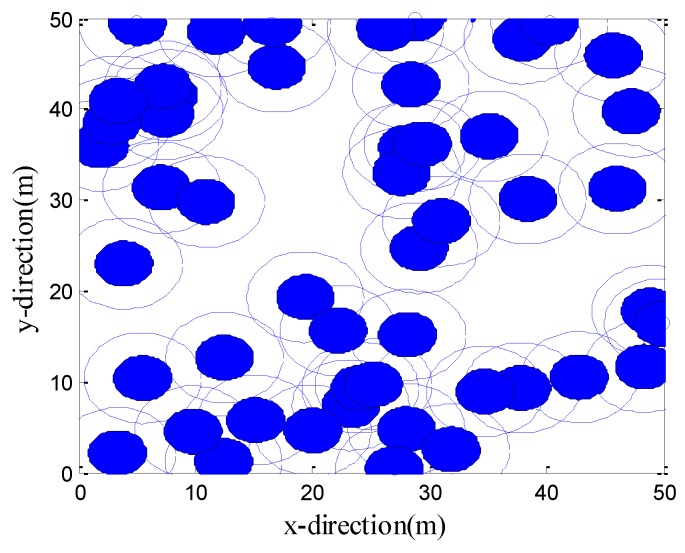
Initial positions of the sensors.

**Figure 4:**
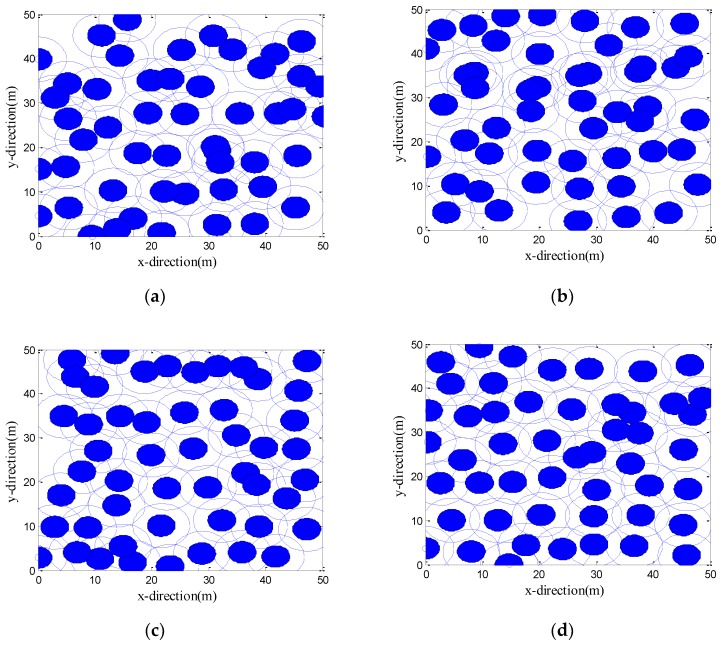
Final deployments of four algorithms. (**a**) Chaos Particle Swarm Optimization (CPSO) algorithm, (**b**) Cuckoo Search (CS) algorithm, (**c**) Lévy-embedded Grey Wolf Optimization (LGWO) algorithm, (**d**) VFLGWO algorithm.

Figure 3 shows the initial positions of the sensor nodes. Figure 4a–d are the development of the best solutions (each large circle represents a schematic representation of the sensing area of the sensor nodes at its center, with a perceived radius *R_s_* = 5 m) after one optimization by the CPSO, CS, LGWO, and VFLGWO algorithms. The coverage rate and uniformity of the CPSO, CS, LGWO, and VFLGWO algorithms for this experiment are listed in Table 4. It can be seen from Figure 4 that the sensor nodes have the most uniform distribution and the largest coverage rate after the VFLGWO algorithm is optimized. The experimental data in Table 4 also confirmed this conclusion.

#### 4.2.3. Multiple Performance Index of Four Algorithms

**Figure 5:**
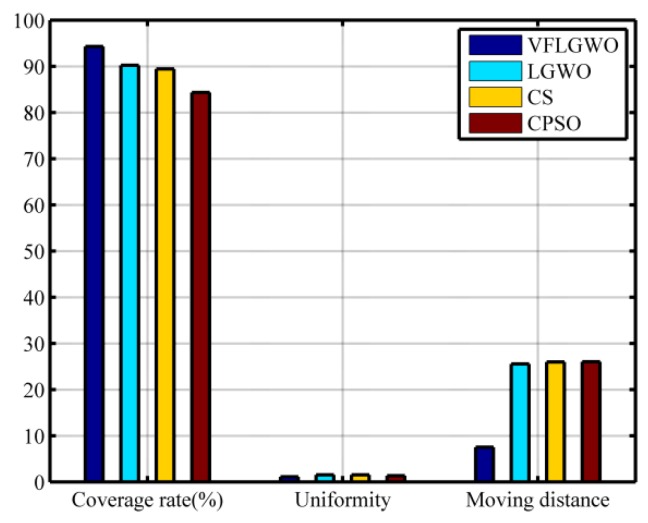
Comparison of the four algorithms.

In this paper, 20 independent experiments of 3000 generations are performed on the four algorithms. The average values of the coverage, uniformity, average moving distance of the sensor nodes, and running time are listed in Table 5. In order to facilitate visual comparison, the data of Table 5 is plotted as a histogram, as shown in Figure 5. It can be seen from Table 5 that the coverage rate of the VFLGWO is improved by 4.05%, 4.87% and 9.94% compared with the other three algorithms, respectively; the uniformity of the VFLGWO algorithm is optimal. The average moving distance of the sensor nodes of the VFLGWO algorithm is significantly shorter than the average moving distance of the sensor nodes of the other three algorithms. In terms of running time of the VFLGWO algorithm, it is about twice that of the other algorithms.

**Figure 6:**
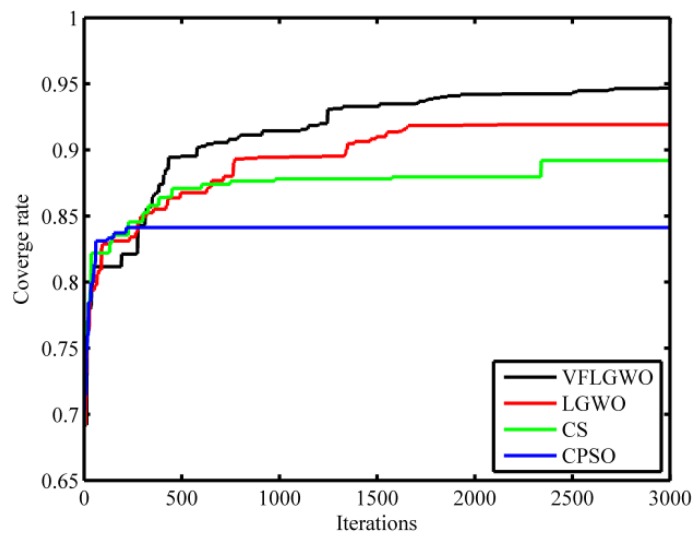
Coverage rate evolution curves of the four algorithms.

Figure 6 shows the network coverage rate curves of the four algorithms, CPSO, CS, LGWO and VFLGWO; running 3000 generations. It can be seen from Figure 6 that the coverage rate of the VFLGWO algorithm is significantly larger than that of the other three algorithms.

### 4.3. Comprehensive Comparison of Experimental Results

#### 4.3.1. Different Numbers of Sensors in the Same Area

**Figure 7:**
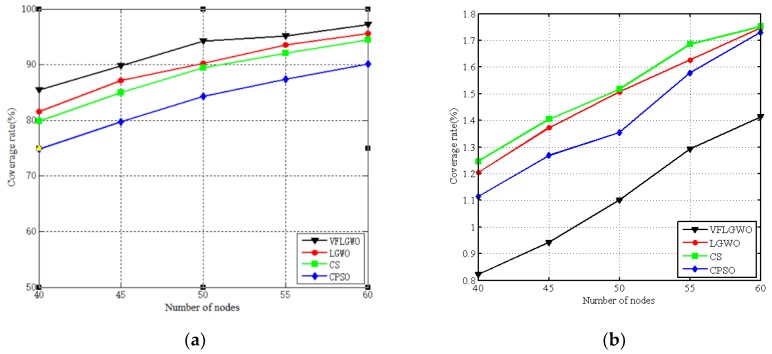

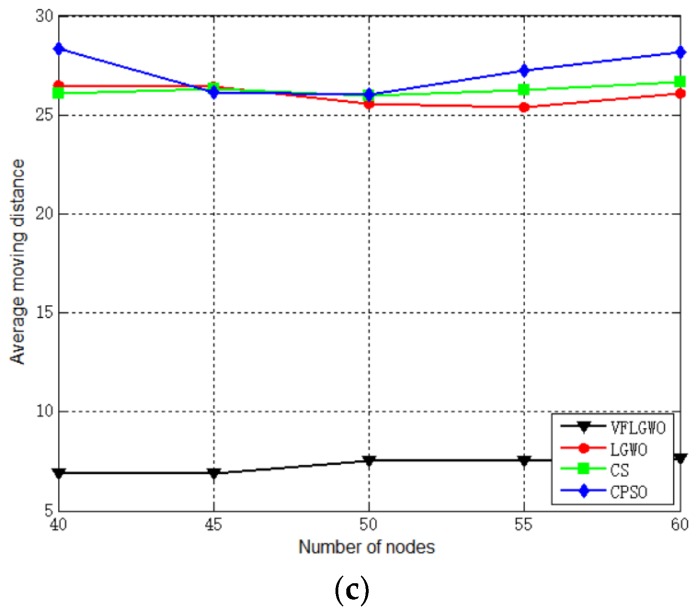
Performance index of the four algorithms with different numbers of sensor nodes. (**a**) Coverage rate of the four algorithms. (**b**) Uniformity of the four algorithms. (**c**) Average moving distance of the four algorithms.

The performance index of the coverage rate, uniformity, and average moving distance of the number of sensor nodes placed in the 50 m × 50 m monitoring area from 40 to 60 (each group adds five sensor nodes) are listed in Table 6, Table 7 and Table 8. In order to facilitate visual comparison, the data of Table 6, Table 7 and Table 8 are plotted as graphs, as shown in Figure 7. It can be seen from Figure 7 that when the size of the monitoring area is the same, the coverage rate of the four algorithms increases as the number of sensor nodes increases. This is because the number of sensor nodes per unit area in the monitoring area increases, and the area coverage rate naturally increases. At the same time, the coverage rate and uniformity of the VFLGWO algorithm are better than the other three algorithms, and the average moving distance of the sensor nodes of the VFLGWO algorithm is significantly shorter than the other three algorithms.

#### 4.3.2. Same Sensor Node Density with Different Monitoring Areas

**Figure 8:**
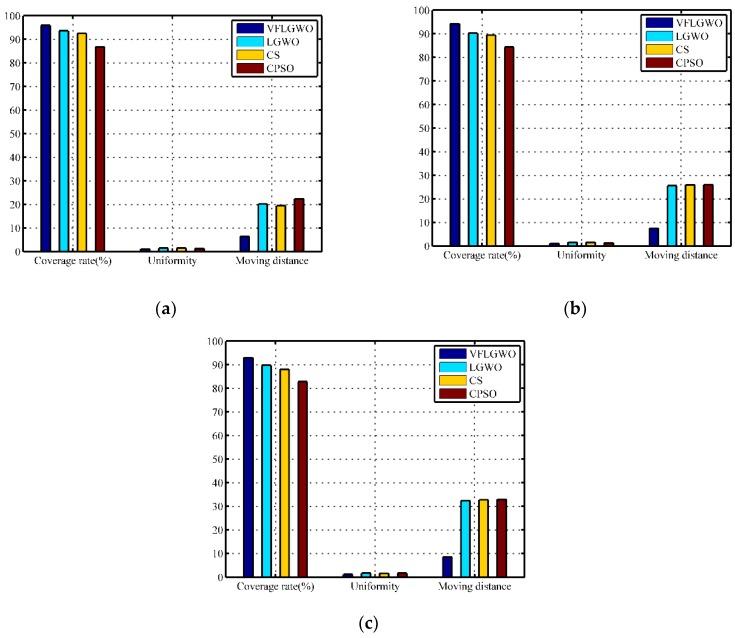
Performance index of four algorithms in different size monitoring areas. (**a**) 40 m × 40 m, (**b**) 50 m × 50 m, (**c**) 60 m × 60 m.

The monitoring area includes 32 sensor nodes in a monitoring area of 40 m × 40 m (50 m^2^/node), and 72 sensor nodes in a monitoring area of 60 m × 60 m (50 m^2^/node). The experimental results are compared with the experimental data of 50 sensor nodes placed in the monitoring area of 50 m × 50 m (50 m^2^/node) in Section 4.3.1. The results are shown in Table 9, Table 10 and Table 11. In order to facilitate visual comparison, the data of Table 9, Table 10 and Table 11 are plotted as histograms, as shown in Figure 8. It can be seen from Figure 8 that the VFLGWO algorithm is superior to the other three algorithms in coverage rate, uniformity, and average moving distance of the sensor nodes in different size monitoring areas.

In summary, coverage rate, uniformity, and average moving distance of sensor nodes of the VFLGWO algorithm achieve a better performance compared to the other three algorithms. The sensor nodes adjusted by the VFLGWO algorithm are more uniformly distributed and have shorter sensor node movement distances. This paper also performs the indicators of the VFLGWO algorithm when the working environment changes. The experimental results of the VFLGWO algorithm are still better than the experimental results of the CPSO, CS and LGWO algorithms.

## 5. Conclusions

In this paper, the modified LGWO algorithm is applied to WSN node deployment. In order to obtain a better performance index, a coverage optimization algorithm for wireless sensor networks with the Virtual Force-Lévy-embedded Grey Wolf Optimization (VFLGWO) algorithm is proposed to solve the coverage optimization problem. The VFLGWO algorithm can effectively make sensor node distribution more uniform, the coverage rate higher in the same environment, the average moving distance of the sensor nodes shorter, and it can ensure the expected design goal is obtained. The simulation results show that the wireless sensor network coverage rate, uniformity, and average moving distance of the VFLGWO algorithm are significantly better than those of the CPSO, CS and LGWO, and the VFLGWO algorithm has good environmental adaptability.

After obtaining the optimal position of the wireless sensor nodes through the VFLGWO algorithm, the sensor nodes matching algorithm proposed in this paper can greatly shorten the average moving distance of the sensor nodes. The running time of the VFLGWO algorithm is relatively long, however because there is no need for frequent scheduling during the deployment of wireless sensor nodes, it will not affect the deployment of WSN nodes—and the coverage rate, uniformity, and average moving distance of the nodes are more important indicators. They affect the regional coverage of the wireless sensor network monitoring area. Our future work will apply the proposed algorithm to the actual scene.

## Figures and Tables

**Table 1:** Four indicators of the Virtual Force-Lévy-embedded Grey Wolf Optimization (VFLGWO) algorithm with different $\omega_{\alpha }$ values.

$\omega_{\gamma }$	Coverage Rate	Uniformity	Moving Distance	Running Time
600	0.9189	1.2521	6.79	1184.5
800	0.93	1.2406	6.95	1133.3
1000	0.9427	1.1014	7.52	1085.8
1500	0.933	1.1412	6.99	1104.5
2000	0.9308	1.0597	6.92	1101.1

**Table 2:** Comparison of four indicators of the VFLGWO algorithm with different *MaxStep* values.

*MaxStep*	Coverage Rate	Uniformity	Moving Distance	Running Time
0.6	0.9273	1.3159	7.33	1050.8
0.8	0.9293	1.2298	7.09	1048.4
1.0	0.9319	1.3202	7.61	1035.7
1.2	0.9427	1.1014	7.52	1085.8
1.4	0.9302	1.2457	7.51	1060.8

**Table 3:** The Chaos Particle Swarm Optimization (CPSO), Cuckoo Search (CS), and Lévy-embedded Grey Wolf Optimization (LGWO) algorithm parameter settings.

Optimizer	Description
CPSO	*c*_1_, *c*_2_ = 2
CS	*p_a_* = 0.25
LGWO	*a*_0_ = 2, *β* ~ *U* (0,2), *p* ~ *U* (0,1)

**Table 4:** Comparison of algorithm coverage and uniformity.

Optimizer	Coverage Rate	Uniformity
CPSO	0.8376	1.3475
CS	0.8856	1.6637
LGWO	0.9108	1.5954
VFLGWO	0.9452	1.1385

**Table 5:** Indicators of the CPSO, CS, LGWO and VFLGWO algorithms for 3000 generations.

Optimizer	Coverage	Uniformity	Moving Distance	Running Time
CPSO	0.8433	1.3549	26.01	513.2
CS	0.894	1.5171	25.98	530.4
LGWO	0.9022	1.5064	25.54	537.1
VFLGWO	0.9427	1.1014	7.52	1085.8

**Table 6:** Coverage rate with different numbers of sensor nodes.

Number of Nodes	40	45	50	55	60
CPSO	0.7478	0.7972	0.8433	0.8744	0.9016
CS	0.7982	0.8501	0.894	0.9201	0.9452
LGWO	0.8153	0.8721	0.9022	0.9358	0.9564
VFLGWO	0.8546	0.8974	0.9427	0.9515	0.9725

**Table 7:** Uniformity with different number of sensor nodes.

Number of Nodes	40	45	50	55	60
CPSO	1.1141	1.268	1.3549	1.5779	1.7299
CS	1.2464	1.4044	1.5171	1.6857	1.7526
LGWO	1.2041	1.3722	1.5064	1.6265	1.7466
VFLGWO	0.8214	0.9419	1.1014	1.2923	1.413

**Table 8:** Average moving distance with different number of sensor nodes.

Number of Nodes	40	45	50	55	60
CPSO	28.32	26.16	26.01	27.26	28.18
CS	26.07	26.29	25.98	26.25	26.67
LGWO	26.47	26.45	25.54	25.36	26.06
VFLGWO	6.91	6.9	7.52	7.53	7.63

**Table 9:** Coverage rate in different size monitoring areas.

Monitoring Area	40 m × 40 m	50 m × 50 m	60 m × 60 m
CPSO	0.8673	0.8433	0.8279
CS	0.9249	0.894	0.8806
LGWO	0.9361	0.9022	0.8979
VFLGWO	0.959	0.9427	0.9292

**Table 10:** Uniformity in different size monitoring areas.

Monitoring Area	40 m × 40 m	50 m × 50 m	60 m × 60 m
CPSO	1.2449	1.3549	1.6919
CS	1.4877	1.5171	1.5977
LGWO	1.4585	1.5064	1.6436
VFLGWO	1.006	1.1014	1.1228

**Table 11:** Average moving distance of sensor nodes in different size monitoring areas.

Monitoring Area	40 m × 40 m	50 m × 50 m	60 m × 60 m
CPSO	22.34	26.01	32.87
CS	19.41	25.98	32.67
LGWO	20.12	25.54	32.41
VFLGWO	6.36	7.52	8.4955
